# Supplementary material for: A proximity-labeling proteomic approach to investigate invadopodia molecular landscape in breast cancer cells
Source: Sci Rep. 2020 Apr 22;10:6787. doi: 10.1038/s41598-020-63926-4 (PMC7176661; doi:10.1038/s41598-020-63926-4)

Supplementary Information

A proximity-labeling proteomic approach to investigate invadopodia molecular landscape in breast cancer cells

Sylvie Thuault[1*], Claire Mamelonet[1], Joëlle Salameh[1,a], Kevin Ostacolo[1,b], Brice Chanez[1,c], Danièle Salaün[1], Emilie Baudelet[2], Stéphane Audebert[2], Luc Camoin[2] and Ali Badache[1]

[1] Centre de Recherche en Cancérologie de Marseille (CRCM), Aix-Marseille Univ, INSERM, Institut Paoli-Calmettes, CNRS, Marseille, France
[2]CRCM, Marseille Proteomics, Aix-Marseille Univ, INSERM, CNRS, Institut Paoli-Calmettes, Marseille, France

Present addresses:
[a] Joëlle Salameh: INSERM UMR-S 1193, Univ. Paris-Sud, Université Paris-Saclay, Châtenay-Malabry, France
[b] Kevin Ostacolo: Department of Biochemistry and Molecular Biology, Biomedical Center, Faculty of Medicine, University of Iceland, Reykjavik, Iceland
[c] Brice Chanez: Institut Paoli-Calmettes, Department of Medical Oncology, Marseille, France

Supplementary Table S1: List of proteins biotinylated by Tks5-BirA* fusion protein. Protein list results from the comparison of proteins biotinylated by Tks5-BirA* fusion protein with proteins biotinylated by BirA* control protein, after their isolation by affinity capture and identification by mass spectrometry. Proteins listed here correspond to proteins above the full line indicative of protein hits obtained at a pFDR of 1% on Fig.3A volcano plot. For each protein their link to invadosome, their implication in actin cytoskeleton organization and their link to cancer invasion and metastasis have been analyzed, by comparison to the list of proteins obtained for "invadosome" query by GLAD4U literature mining and manual literature searching. The presence of Proline-rich regions and SH3 domains determined by InterPro site is also referenced. Data result from two different experiments processed three times.

Supplementary Table S2: List of proteins biotinylated by ΔPX-Tks5-BirA* fusion protein. Protein list results from the comparison of proteins biotinylated by ΔPX-Tks5-BirA* fusion protein with proteins biotinylated by BirA* control protein, after their isolation by affinity capture and identification by mass spectrometry. Proteins listed here correspond to proteins hits obtained at a pFDR of 1%. Data result from two different experiments processed three times.

Supplementary Table S3: Tables listing proteins biotinylated by both Tks5-BirA* and ΔPX-Tks5-BirA* (left), only by Tks5-BirA* (middle) and only by ΔPX-Tks5-BirA* (right) fusion proteins. Proteins listed here correspond to proteins highlighted in red, blue and green, respectively, on Fig.4A volcano plot. Tables result from the intersection of Tks5-BirA* (Supplementary Table S1) and ΔPX-Tks5-BirA* (Supplementary Table S2) close neighbors. Proteins biotinylated by both Tks5-BirA* fusion proteins correspond to proteins identified as different (pFDR of 1%) in each of the fusion protein relative to BirA* control condition. Proteins designed as Tks5-BirA* or ΔPX-Tks5-BirA* only correspond to proteins different (pFDR of 1%) only in the fusion protein addressed relative to BirA* control condition. For each protein, its difference of abundance in Tks5-BirA* fusion proteins *versus* BirA* conditions is indicated and its difference of abundance in Tks5-BirA* *versus* ΔPX-Tks5-BirA* conditions has been calculated and is reported in the third column. (1) Grey-colored columns correspond to proteins with no significant differential of abundance in the Tks5-BirA* fusion protein analyzed relative to BirA* control condition.

Supplementary Table S4: Primers pairs used for amplifying Tks5 and ΔPX-Tks5 sequences.

Supplementary Figure 1: (A) Analysis of the efficiency of Tks5 knockdown by western blotting. MDA-MB-231 cells stably expressing BirA*, Tks5-BirA* or ΔPX-Tks5-BirA* were transfected with a siRNA directed against the 3'UTR of Tks5 mRNA (siTks5) or with a control siRNA directed against LacZ mRNA (siLacZ) for 36 h. An anti-Tks5 antibody was used to analyze Tks5 levels. α-Tubulin was used as a loading control. The efficiency of Tks5 knockdown is clearly observed in BirA* expressing cells. In cells expressing Tks5-BirA* fusion proteins, degradation products coincided with the size of endogenous Tks5 making it difficult to visualize the efficiency of Tks5 knock-down in these cells. **(B) Analysis of the biotinylation of newly identified Tks5 neighbors in presence or absence of endogenous Tks5.** MDA-MB-231 cells stably expressing BirA*, Tks5-BirA* or ΔPX-Tks5-BirA* were transfected with a siRNA against the 3'UTR of Tks5 mRNA (siTks5) or with a siRNA control directed against LacZ mRNA (siLacZ) for 36 h and then seeded on gelatin in presence of biotin for 16 h. After cell lysis, biotinylated proteins were isolated by affinity capture using avidin-coated beads. The presence of proteins of interest was assessed in whole cell extract (WCE) or after avidin-based pulldown by western blotting with specific antibodies. Knock-down of endogenous Tks5 had no impact on the efficiency of Tks5-BirA fusion proteins to biotinylate newly identified Tks5 close neighbors. Full-length blots are presented in Supplementary Figure 2.

Supplementary Figure 2: Full-size blots/gels from Figures 1C, 2B-D, 4B and Supplementary Figure 1.

Supplementary Table S1: List of proteins biotinylated by Tks5-BirA* fusion protein.

Protein list results from the comparison of proteins biotinylated by Tks5-BirA* fusion protein with proteins biotinylated by BirA* control protein, after their isolation by affinity capture and identification by mass spectrometry.
Proteins listed here correspond to proteins above the full line indicative of protein hits obtained at a pFDR of 1% on Fig.3A volcano plot.
For each protein their link to invadosome, their implication in actin cytoskeleton organization and their link to cancer invasion and metastasis have been analyzed, by comparison to the list of proteins obtained for "invadosome" query by GLAD4U literature mining and manual literature searching.
The presence of Proline-rich regions and SH3 domains determined by InterPro site is also referenced.
Data result from two different experiments processed three times.

Protein IDs	Protein names	Gene names	Peptides	Sequence coverage [%]	Mol. weight [kDa]	p-value (-Log10)	Differential (Log2) Tks5-BirA* *versus* BirA*	Linked to invadosomes	Implicated in actin cytoskeleton reorganization	Linked to cancer invasion/metastasis	SH3 containing proteins	Proline-rich region containing proteins	References (PMID:)
Q5TCZ1	SH3 and PX domain-containing protein 2A	SH3PXD2A	120	81	125.29	13.91414333	16.10223103	Yes	Yes	Yes	Yes	Yes	
P49959	Double-strand break repair protein MRE11A	MRE11A	18	35.9	80.592	7.93361717	7.613502502			Yes			22914783; 30176843
Q14247	Src substrate cortactin	CTTN	28	50.5	61.585	10.4023223	7.51793925	Yes	Yes	Yes	Yes		10442635; 29172953
Q9NQC3	Reticulon-4	RTN4	7	8.6	129.93	7.876353516	7.337134679		Yes	Yes		Yes	28628795; 23042479; 27132996
Q9Y5K6	CD2-associated protein	CD2AP	13	24.3	71.45	8.983150317	7.225888411	(Yes)	Yes		Yes	Yes	10339567; 23090967
O60732	Melanoma-associated antigen C1	MAGEC1	6	8.5	123.64	5.386851732	5.631188869			Yes			21738656; 17607369
P98174	FYVE, RhoGEF and PH domain-containing protein 1	FGD1	7	12.3	106.56	8.73033328	4.930959066	Yes	Yes	Yes		Yes	19141649; 22854039
P27816	Microtubule-associated protein 4	MAP4	16	20.4	121	8.687608396	4.929625193		Yes	Yes		Yes	21937675;24140250; 26876215
Q96RU3	Formin-binding protein 1	FNBP1	6	12.5	71.306	4.260582509	4.438922723	Yes	Yes	Yes	Yes	Yes	29651632; 21421245
P29353	SHC-transforming protein 1	SHC1	4	16.8	62.821	6.160669832	3.875651677		Yes	Yes		Yes	24078705; 18604176; 23277357
Q14195	Dihydropyrimidinase-related protein 3	DPYSL3	12	30.4	61.963	3.494760306	3.636353652		Yes	Yes (+ or -)			16181627; 26934554; 22805864; 20543870
Q9Y6M1	Insulin-like growth factor 2 mRNA-binding protein 2	IGF2BP2	3	8.5	66.121	4.416141395	2.985394319	Yes		Yes			29217458; 16541107; 23069990
Q9NS25	Sperm protein associated with the nucleus on the X chromosome B/F	SPANXB1	2	27.2	11.84	6.075416422	2.941437085			Yes (+ or -)			26895102; 31406142; 27323831
Q9NS26	Sperm protein associated with the nucleus on the X chromosome A	SPANXA1											
Q86YT6	E3 ubiquitin-protein ligase MIB1	MIB1	2	3.4	110.13	3.916397356	2.915704568		Yes				29078376
Q8TE02	Elongator complex protein 5	ELP5	5	24.1	34.841	3.2596053	2.9046731			Yes			22854966
Q9UKK9	ADP-sugar pyrophosphatase	NUDT5	4	23.3	24.327	3.925240339	2.815107028						
Q96EB1	Elongator complex protein 4	ELP4	6	24.1	46.587	7.643664097	2.812804858			Yes			29805303
Q9H6X2	Anthrax toxin receptor 1	ANTXR1	4	11.5	62.788	3.883642755	2.784154256		Yes	Yes		Yes	19817382; 23832666
O00151	PDZ and LIM domain protein 1	PDLIM1	4	27.7	36.071	3.126147395	2.704363346		Yes	Yes (+ or -)			11110697; 31509262; 26701804; 24662836
Q13444	Disintegrin and metalloproteinase domain-containing protein 15	ADAM15	3	5.6	92.958	4.679859557	2.634385427	(Yes)		Yes (+ or -)		Yes	26930657; 18281484; 20851104
Q7Z6K5	Arpin	ARPIN	1	5.3	24.943	6.153299742	2.345861912		Yes	Yes (-)			24132237; 30416079; 26867158
Q70E73	Ras-associated and pleckstrin homology domains-containing protein 1	RAPH1	3	4.7	135.25	2.33985344	2.165382385	Yes	Yes	Yes		Yes	26996666; 30056565
Q9UJU6	Drebrin-like protein	DBNL	1	2.6	48.207	3.83907753	2.131621997	Yes	Yes	Yes (+ or -)	Yes		27129278; 22303001; 24912570
Q07157	Tight junction protein ZO-1	TJP1	24	20.7	195.46	7.2425774	2.101765315	Yes	Yes	Yes ?	Yes	Yes	29765546; 20930113
P55209	Nucleosome assembly protein 1-like 1	NAP1L1	2	5.4	45.374	3.573022604	2.077949206			Yes			31516385
Q99733	Nucleosome assembly protein 1-like 4	NAP1L4											
O43426	Synaptojanin-1	SYNJ1	2	3.1	173.1	4.141125533	2.071942647	(Yes)				Yes	25605973; 9199318
Q99661	Kinesin-like protein KIF2C	KIF2C	2	5	81.312	2.094197001	1.995908578			Yes			18506187; 17653072; 31073307
Q13162	Peroxiredoxin-4	PRDX4	2	8.1	30.54	2.563453061	1.873274485			Yes			31311441; 21487000; 25054044
O75815	Breast cancer anti-estrogen resistance protein 3	BCAR3	2	3.3	92.565	2.254819159	1.872347673		Yes	Yes (+/-)			25499443; 17616674; 23762409
P50479	PDZ and LIM domain protein 4	PDLIM4	2	10.6	35.398	2.950149833	1.868245602		Yes	Yes (-)			14729062; 31376371; 20642860
P10398	Serine/threonine-protein kinase A-Raf	ARAF	1	3	67.585	2.441532048	1.833749294						
Q0PNE2	Elongator complex protein 6	ELP6	2	9.8	29.793	2.127950462	1.777745883			Yes			22854966
P11908	Ribose-phosphate pyrophosphokinase 2	PRPS2	8	36.2	34.769	5.812583924	1.750796954			Yes			30908912
Q9Y4E8	Ubiquitin carboxyl-terminal hydrolase 15	USP15	1	2.1	112.42	2.472299838	1.748913447						
P38919	Eukaryotic initiation factor 4A-III;Eukaryotic initiation factor 4A-III, N-terminally processed	EIF4A3	13	29.7	46.871	7.984805868	1.744141579						
Q9UPQ0	LIM and calponin homology domains-containing protein 1	LIMCH1	2	2.7	121.87	3.924842063	1.579572042		Yes				28228547
P41091	Eukaryotic translation initiation factor 2 subunit 3	EIF2S3	9	30.3	51.109	7.34536761	1.521572113						
Q2VIR3	Putative eukaryotic translation initiation factor 2 subunit 3-like protein	EIF2S3L											
P49247	Ribose-5-phosphate isomerase	RPIA	5	22.5	33.269	6.120177273	1.411331177			Yes			25429733
Q9UDY2	Tight junction protein ZO-2	TJP2	14	17.2	133.96	7.114464632	1.4007864	(Yes)	Yes	Yes (-)	Yes		23605953
P49321	Nuclear autoantigenic sperm protein	NASP	4	11.4	85.237	6.253539977	1.394634247						
P0DMV9	Heat shock 70 kDa protein 1B	HSPA1B	37	70.2	70.051	9.848124347	1.286704063			Yes			19914824; 20564126; 25213699
P0DMV8	Heat shock 70 kDa protein 1A	HSPA1A											

Supplementary Table S2: List of proteins biotinylated by ∆PX-Tks5-BirA* fusion protein.

Protein list results from the comparison of proteins biotinylated by ∆PX-Tks5-BirA* fusion protein with proteins biotinylated by BirA* control protein, after their isolation by affinity capture and identification by mass spectrometry.
Proteins listed here correspond to proteins hits obtained at a pFDR of 1%. Data result from two different experiments processed three times.

Protein IDs	Protein names	Gene names	Peptides	Sequence coverage [%]	Mol. weight [kDa]	p-value (-Log10)	Differential (Log2) ∆PX-Tks5-BirA* *versus* BirA*
Q5TCZ1;A1X283	SH3 and PX domain-containing protein 2A	SH3PXD2A	120	81	125.29	14.00469377	16.79133638
Q14247	Src substrate cortactin	CTTN	28	50.5	61.585	10.5672796	7.658259074
P49959	Double-strand break repair protein MRE11A	MRE11A	18	35.9	80.592	7.46094941	7.174569766
Q9Y5K6	CD2-associated protein	CD2AP	13	24.3	71.45	7.966618016	6.196915785
Q96RU3	Formin-binding protein 1	FNBP1	6	12.5	71.306	4.670416997	5.608518759
O60732	Melanoma-associated antigen C1	MAGEC1	6	8.5	123.64	5.166906572	5.325631301
Q14195	Dihydropyrimidinase-related protein 3	DPYSL3	12	30.4	61.963	4.583939755	5.090364933
Q86T24	Transcriptional regulator Kaiso	ZBTB33	3	8.8	74.484	7.226418622	4.578668594
Q969G3	SWI/SNF-related matrix-associated actin-dependent regulator of chromatin subfamily E member 1	SMARCE1	2	8	46.649	2.205633135	4.180418491
Q15652	Probable JmjC domain-containing histone demethylation protein 2C	JMJD1C	3	1.8	284.52	5.743897208	4.147589048
Q9Y6M1	Insulin-like growth factor 2 mRNA-binding protein 2	IGF2BP2	3	8.5	66.121	5.538307203	3.866715272
Q96EB1	Elongator complex protein 4	ELP4	6	24.1	46.587	8.216873652	3.530961037
O00151	PDZ and LIM domain protein 1	PDLIM1	4	27.7	36.071	3.974932048	3.492241383
Q9UKK9	ADP-sugar pyrophosphatase	NUDT5	4	23.3	24.327	4.395425637	3.363261541
Q8TE02	Elongator complex protein 5	ELP5	5	24.1	34.841	3.549250869	3.296580474
P50479	PDZ and LIM domain protein 4	PDLIM4	2	10.6	35.398	4.918884984	3.1442132
Q7Z6K5	Arpin	ARPIN	1	5.3	24.943	5.78745232	3.105995337
P31946	14-3-3 protein beta/alpha;14-3-3 protein beta/alpha, N-terminally processed	YWHAB	6	40.7	28.082	3.102797521	2.770226796
Q5T0N5	Formin-binding protein 1-like	FNBP1L	3	8.6	70.065	3.163598993	2.732745806
P61981	14-3-3 protein gamma;14-3-3 protein gamma, N-terminally processed	YWHAG	5	32.4	28.302	3.841400861	2.722501755
P27816	Microtubule-associated protein 4	MAP4	16	20.4	121	6.926183764	2.682752927
Q9NS25	Sperm protein associated with the nucleus on the X chromosome B/F	SPANXB1	2	27.2	11.84	5.73212081	2.642450651
Q9NS26	Sperm protein associated with the nucleus on the X chromosome A	SPANXA1					
Q07157	Tight junction protein ZO-1	TJP1	24	20.7	195.46	8.285232815	2.545808792
Q0PNE2	Elongator complex protein 6	ELP6	2	9.8	29.793	2.754979228	2.469645182
Q9Y4E8	Ubiquitin carboxyl-terminal hydrolase 15	USP15	1	2.1	112.42	3.096935139	2.253142675
O43426	Synaptojanin-1	SYNJ1	2	3.1	173.1	4.567370702	2.237295151
P29401	Transketolase	TKT	4	11.1	67.877	2.172138733	2.136732419
P55209	Nucleosome assembly protein 1-like 1	NAP1L1	2	5.4	45.374	3.424767194	2.123708725
Q99733	Nucleosome assembly protein 1-like 4	NAP1L4					
Q9HCN4	GPN-loop GTPase 1	GPN1	1	4.3	41.74	3.390386248	2.03527228
P23588	Eukaryotic translation initiation factor 4B	EIF4B	2	6.2	69.15	2.576545034	1.942392349
O75815	Breast cancer anti-estrogen resistance protein 3	BCAR3	2	3.3	92.565	2.435325241	1.941552321
P62258	14-3-3 protein epsilon	YWHAE	9	37.6	29.174	7.019493727	1.934883118
Q9UJU6	Drebrin-like protein	DBNL	1	2.6	48.207	1.841725873	1.888232867
Q9UDY2	Tight junction protein ZO-2	TJP2	14	17.2	133.96	8.786069397	1.886053403
Q9UPQ0	LIM and calponin homology domains-containing protein 1	LIMCH1	2	2.7	121.87	4.156091474	1.855589549
P40763	Signal transducer and activator of transcription 3	STAT3	1	3.4	88.067	2.973856904	1.832819303
Q13162	Peroxiredoxin-4	PRDX4	2	8.1	30.54	2.654100435	1.831843694
Q9UHB6	LIM domain and actin-binding protein 1	LIMA1	4	7.4	85.225	3.11598462	1.791516304
P0DMV9	Heat shock 70 kDa protein 1B	HSPA1B	37	70.2	70.051	8.983589311	1.561729113
P0DMV8	Heat shock 70 kDa protein 1A	HSPA1A					
P49023	Paxillin	PXN	8	23.2	64.505	5.453557229	1.528698921
P63104	14-3-3 protein zeta/delta	YWHAZ	10	45.3	27.745	4.353593263	1.456914902
P34931	Heat shock 70 kDa protein 1-like	HSPA1L	15	29.8	70.374	4.714809086	1.392109235
O60711	Leupaxin	LPXN	1	5.7	43.332	4.515592353	1.385031064
P49321	Nuclear autoantigenic sperm protein	NASP	4	11.4	85.237	6.546653084	1.354332606
Q09666	Neuroblast differentiation-associated protein AHNAK	AHNAK	104	39.7	629.09	6.490538917	1.249121666

Supplementary Table S3: Tables listing proteins biotinylated by both Tks5-BirA* and ΔPX-Tks5-BirA*(left), only by Tks5-BirA* (middle) and only by ΔPX-Tks5-BirA* (right) fusion proteins.

Proteins listed here correspond to proteins highlighted in red, blue and green, respectively, on Fig.4A volcano plot
Tables result from the intersection of Tks5-BirA* (Supplementary Table S1) and ΔPX-Tks5-BirA* (Supplementary Table S2) close neighbors.
Proteins biotinylated by both Tks5-BirA* fusion proteins correspond to proteins identified as different (pFDR < 1%) in each of the fusion protein relative to BirA* control condition.
Proteins designed as Tks5-BirA* or DPX-Tks5-BirA* only correspond to proteins different (pFDR < 1%) only in the fusion protein addressed relative to BirA* control condition.
For each protein, its difference of abundance in Tks5-BirA* fusion proteins versus BirA* conditions is indicated, and its difference of abundance in Tks5-BirA* versus DPX-Tks5-BirA* conditions has been calclutated and is reported in the third column.
(1) Grey-colored columns correspond to proteins with no significant differential of abundance in the Tks5-BirA* fusion protein analyzed relative to BirA* control condition.

In both (27)	Differential (Log2) Tks5-BirA* *versus* BirA*	Differential (Log2) ΔPX-Tks5-BirA* *versus* BirA*	Differential (Log2) Tks5-BirA* *versus* ΔPX-Tks5-BirA*
MAP4	4.93	2.68	2.25
CD2AP	7.23	6.20	1.03
MRE11A	7.61	7.17	0.44
MAGEC1	5.63	5.33	0.31
SPANXA1/SPANXB1	2.94	2.64	0.30
DBNL	2.13	1.89	0.24
PRDX4	1.87	1.83	0.04
NASP	1.39	1.35	0.04
NAP1L1/NAP1L4	2.08	2.12	-0.05
BCAR3	1.87	1.94	-0.07
CTTN	7.52	7.66	-0.14
SYNJ1	2.07	2.24	-0.17
HSPA1A/HSPA1B	1.29	1.56	-0.28
LIMCH1	1.58	1.86	-0.28
ELP5	2.90	3.30	-0.39
TJP1	2.10	2.55	-0.44
TJP2	1.40	1.89	-0.49
USP15	1.75	2.25	-0.50
NUDT5	2.82	3.36	-0.55
ELP6	1.78	2.47	-0.69
ELP4	2.81	3.53	-0.72
ARPIN	2.35	3.11	-0.76
PDLIM1	2.70	3.49	-0.79
IGF2BP2	2.99	3.87	-0.88
FNBP1	4.44	5.61	-1.17
PDLIM4	1.87	3.14	-1.28
DPYSL3	3.64	5.09	-1.45

In Tks5-BirA* only (13)	Differential (Log2) Tks5-BirA* *versus* BirA*	Differential (Log2) ΔPX-Tks5-BirA* *versus* BirA* (1)	Differential (Log2) Tks5-BirA* *versus* ΔPX-Tks5-BirA*
RTN4	7.34	1.74	5.60
FGD1	4.93	0.00	4.93
SHC1	3.88	1.47	2.40
MIB1	2.92	2.64	0.27
ANTXR1	2.78	1.15	1.64
ADAM15	2.63	-0.38	3.01
RAPH1	2.17	1.44	0.72
KIF2C	2.00	1.11	0.88
ARAF	1.83	1.22	0.61
PRPS2	1.75	1.30	0.45
EIF4A3	1.74	-0.47	2.21
EIF2S3/EIF2S3L	1.52	1.16	0.36
RPIA	1.41	1.10	0.31

In ΔPX-Tks5-BirA* only (17)	Differential (Log2) Tks5-BirA* *versus* BirA* (1)	Differential (Log2) ΔPX-Tks5–BirA* *versus* BirA*	Differential (Log2) Tks5-BirA* *versus* ΔPX-Tks5-BirA*
ZBTB33	0.86	4.58	-3.72
SMARCE1	0.00	4.18	-4.18
JMJD1C	1.13	4.15	-3.02
YWHAB	0.22	2.77	-2.55
FNBP1L	1.36	2.73	-1.38
YWHAG	0.22	2.72	-2.50
TKT	1.54	2.14	-0.59
GPN1	0.64	2.04	-1.39
EIF4B	1.51	1.94	-0.43
YWHAE	0.64	1.93	-1.29
STAT3	1.15	1.83	-0.68
LIMA1	1.31	1.79	-0.48
PXN	1.16	1.53	-0.37
YWHAZ	0.35	1.46	-1.11
HSPA1L	1.06	1.39	-0.33
LPXN	0.77	1.39	-0.62
AHNAK	1.18	1.25	-0.07

Supplementary Table S4 : Primer pairs used for amplifying Tks5 and ΔPX-Tks5 sequences

Name	Sequence
attB1-Nter Tks5	5’-GGGGACAAGTTTGTACAAAAAAGCAGGCTTCACCATGCTCGC CTACTGCGTGCAGGATG-3’
attB1-delPX Tks5	5’-GGGGACAAGTTTGTACAAAAAAGCAGGCTTCACCATGGAGGC TCGACCCGAGGATGTC-3’
attB2-Cter Tks5	5’-GGGGACCACTTTGTACAAGAAAGCTGGGTCGTTCTTTTTCTCAAGGTAGTTGGAAG-3’

Thuault et al., Supplementary Figure 1

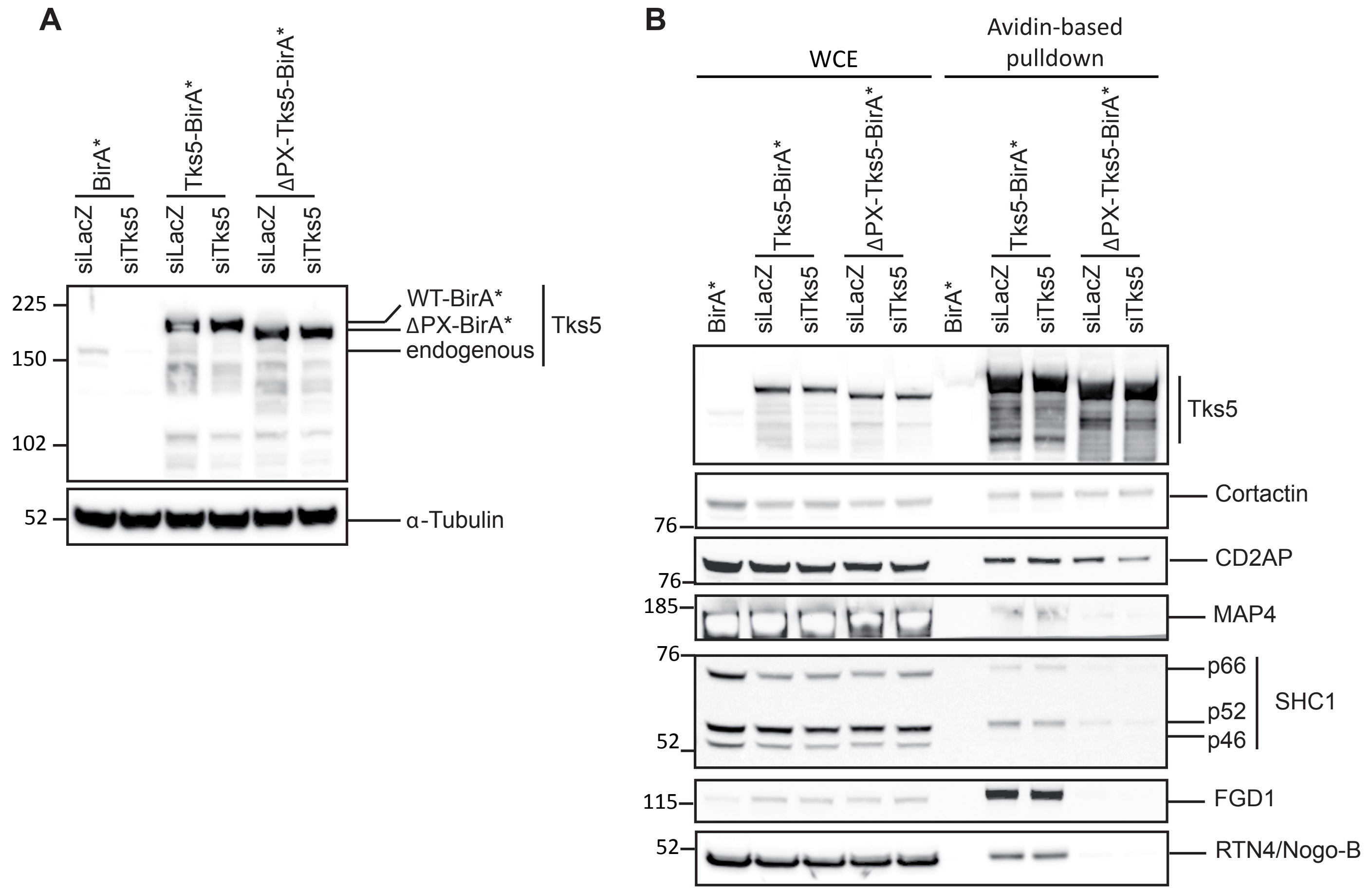

Thuault et al., Supplementary Figure 2

Figure 1C

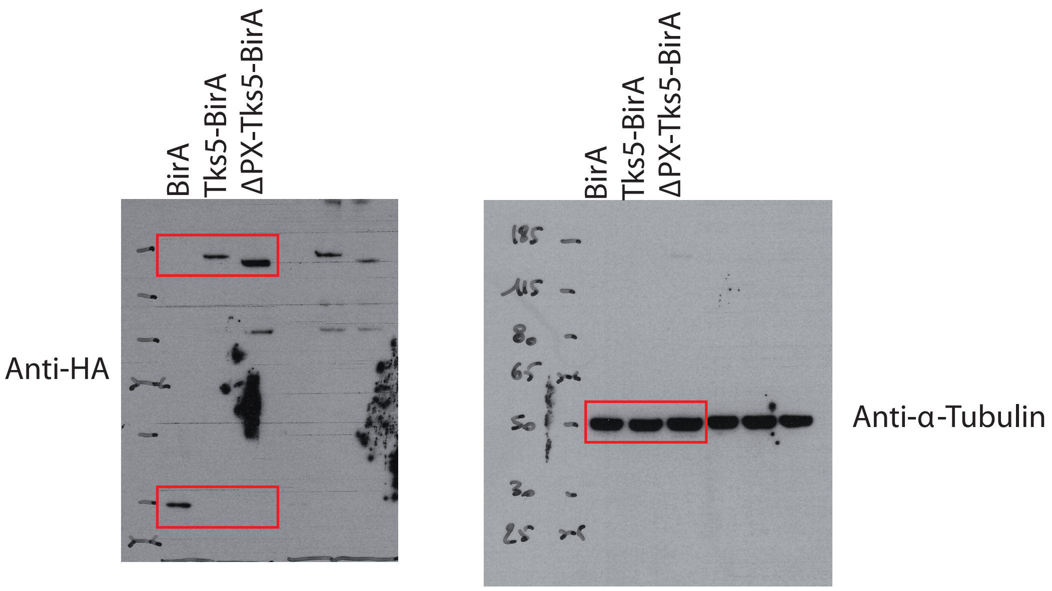

Figure 2B

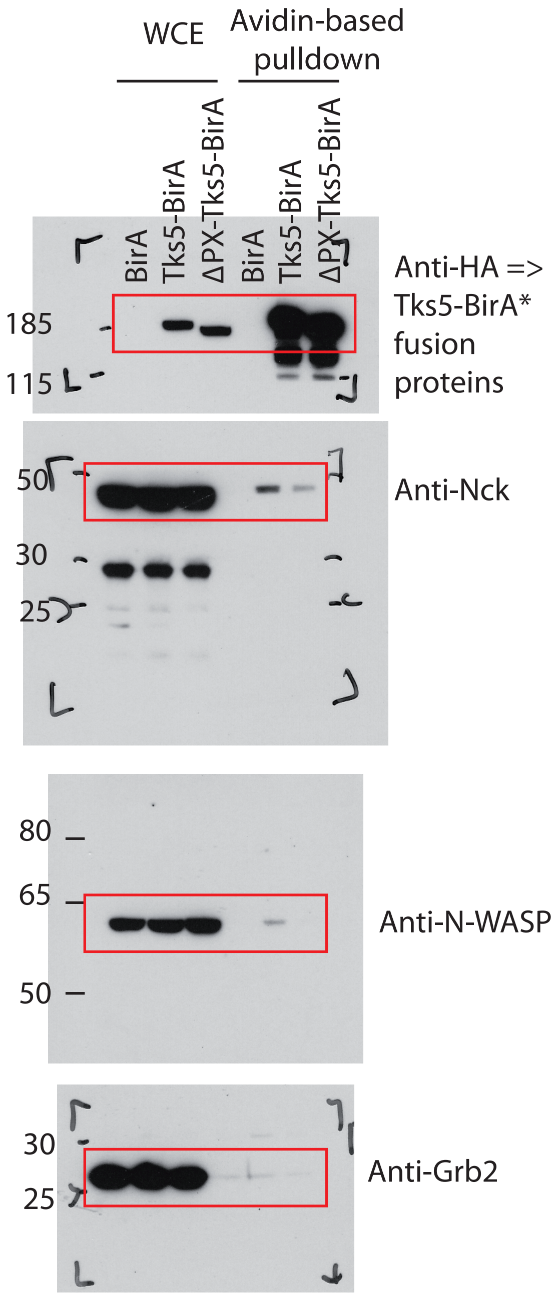

Figure 2C

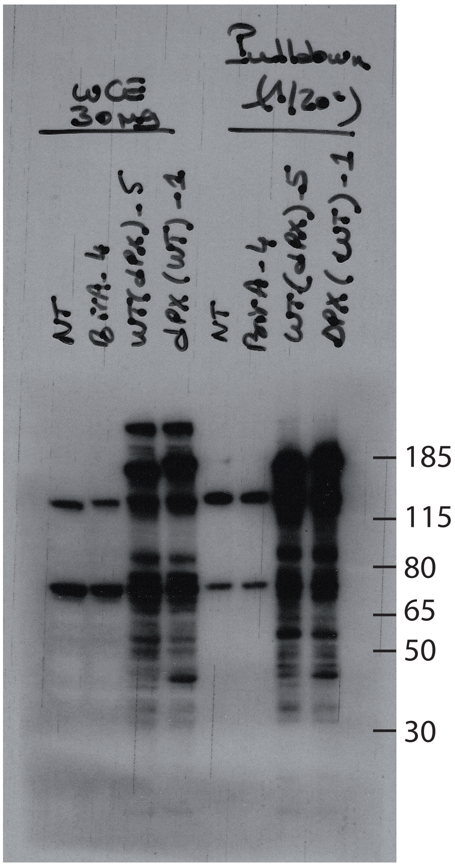

Figure 2D

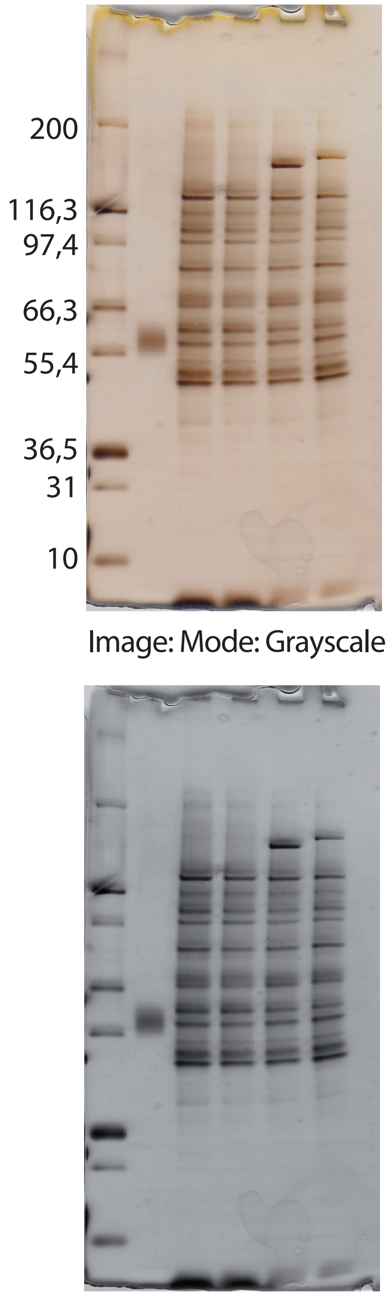

Figure 4B

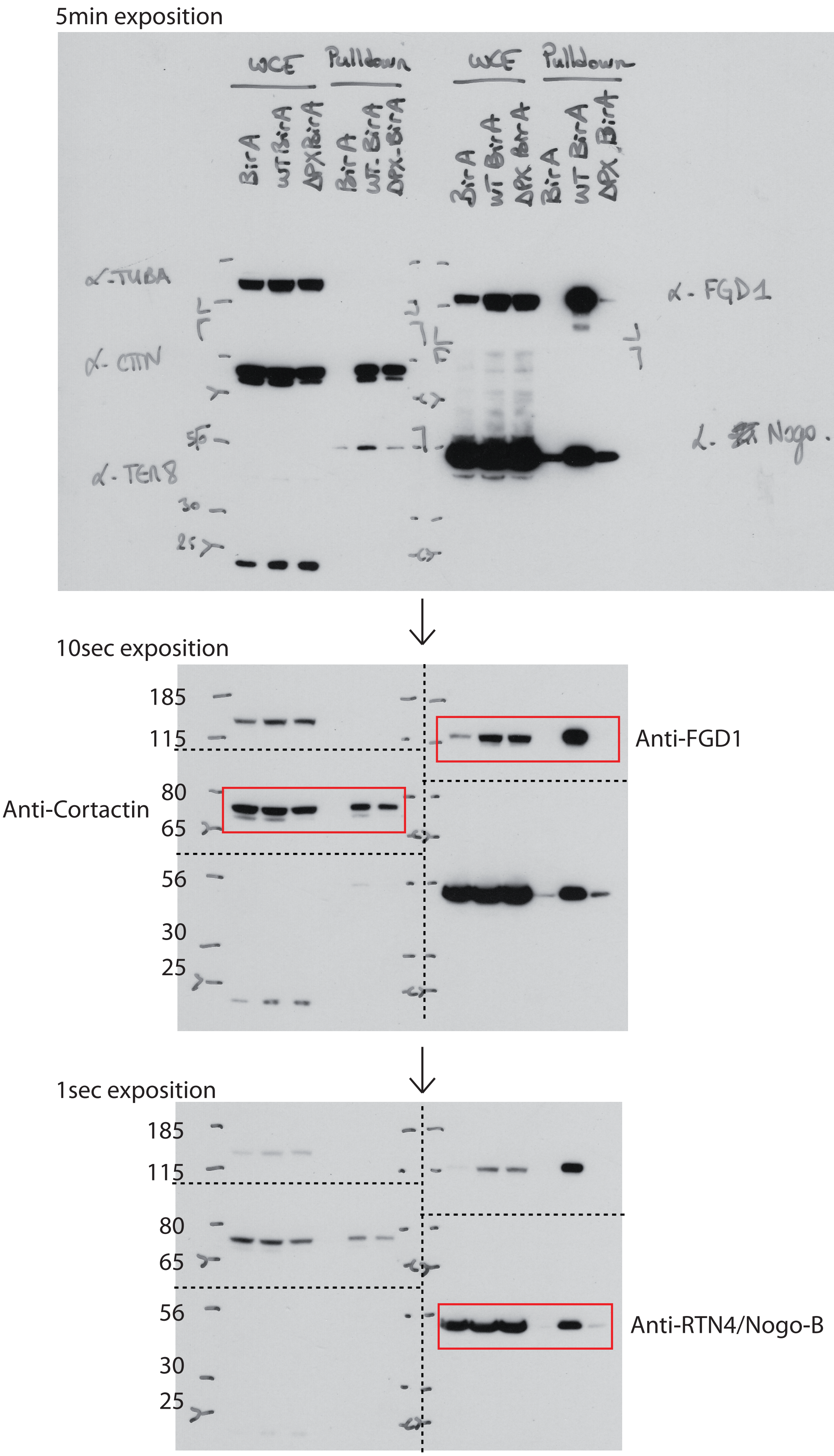

5min exposition
WCE
Pulldown
BirA
WT BirA
ΔPX BirA
α-TUBA
α-CTTN
α-TEN8
α-FGD1
α-Nogo
10sec exposition
185
115
80
65
56
30
25
Anti-Cortactin
Anti-FGD1
1sec exposition
Anti-RTN4/Nogo-B

Figure 4B (continuation)

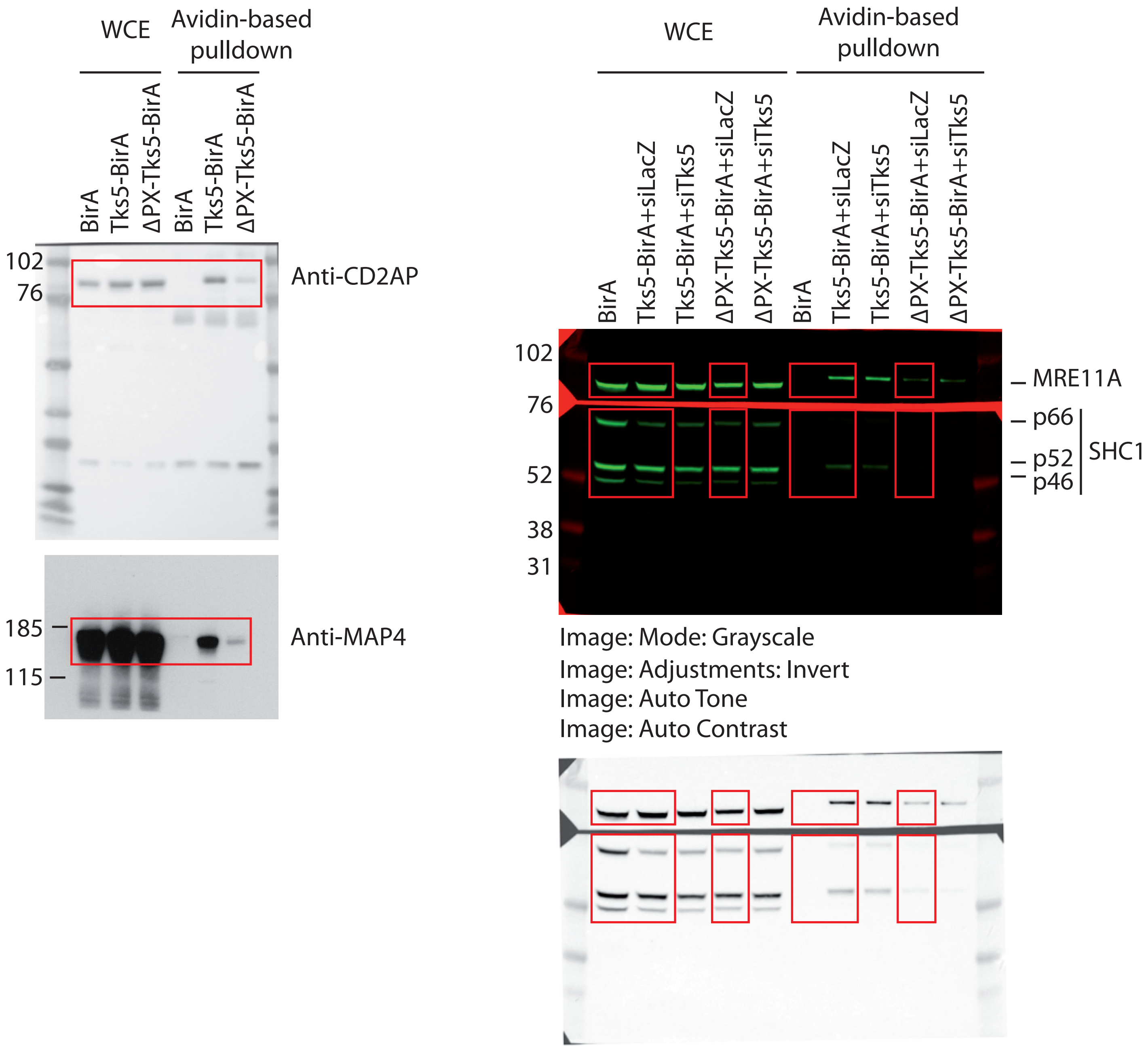

Supplementary Figure 1

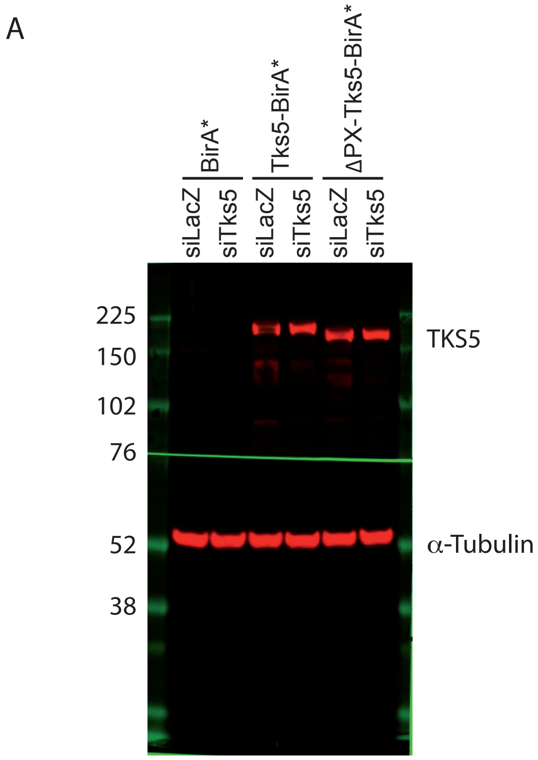

Image: Mode: Grayscale
Image: Adjustments: Invert
Image: Auto Tone
Image: Auto Contrast

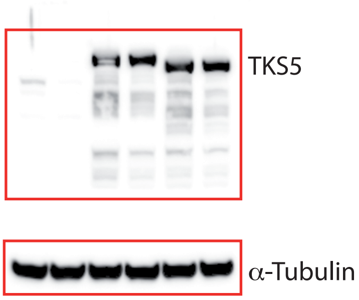

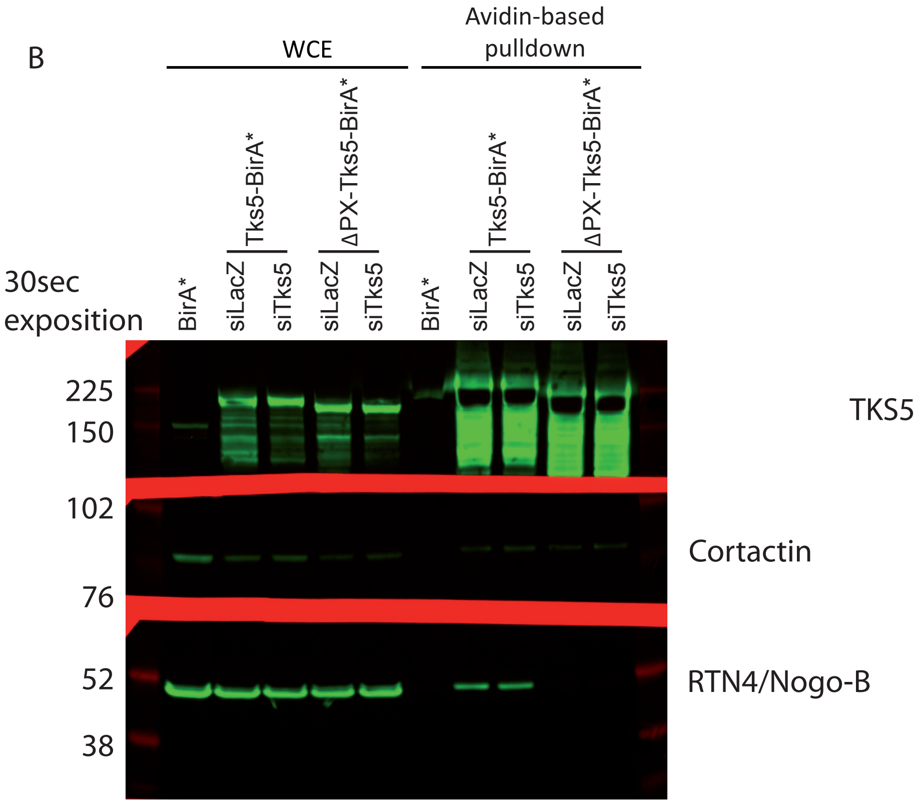

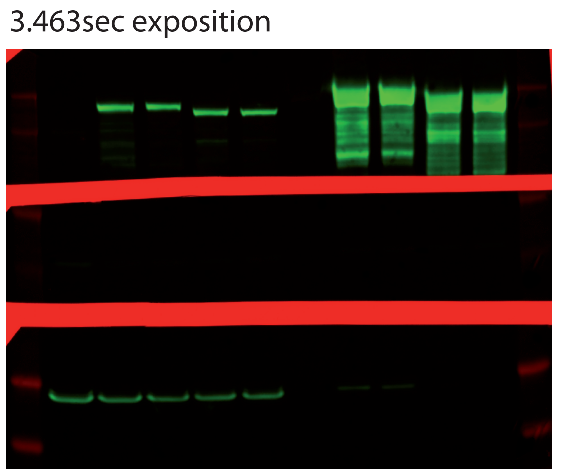

Image: Mode: Grayscale
Image: Adjustments: Invert
Image: Auto Tone
Image: Auto Contrast

Image: Mode: Grayscale
Image: Adjustments: Invert
Image: Auto Tone
Image: Auto Contrast

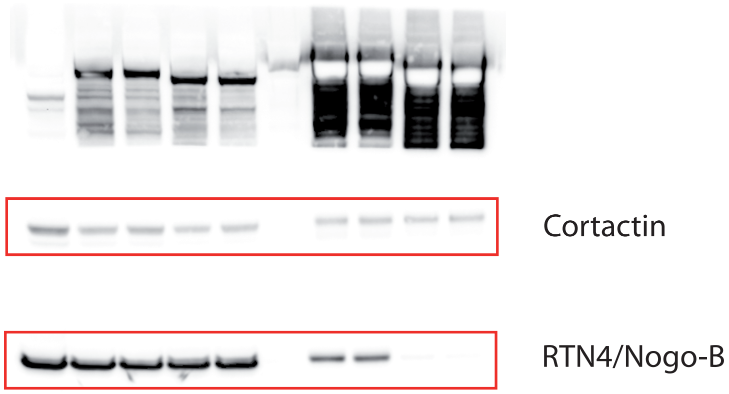

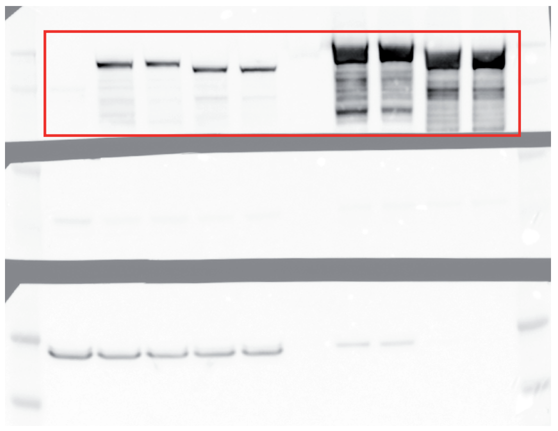

Supplementary Figure 1 (continuation)

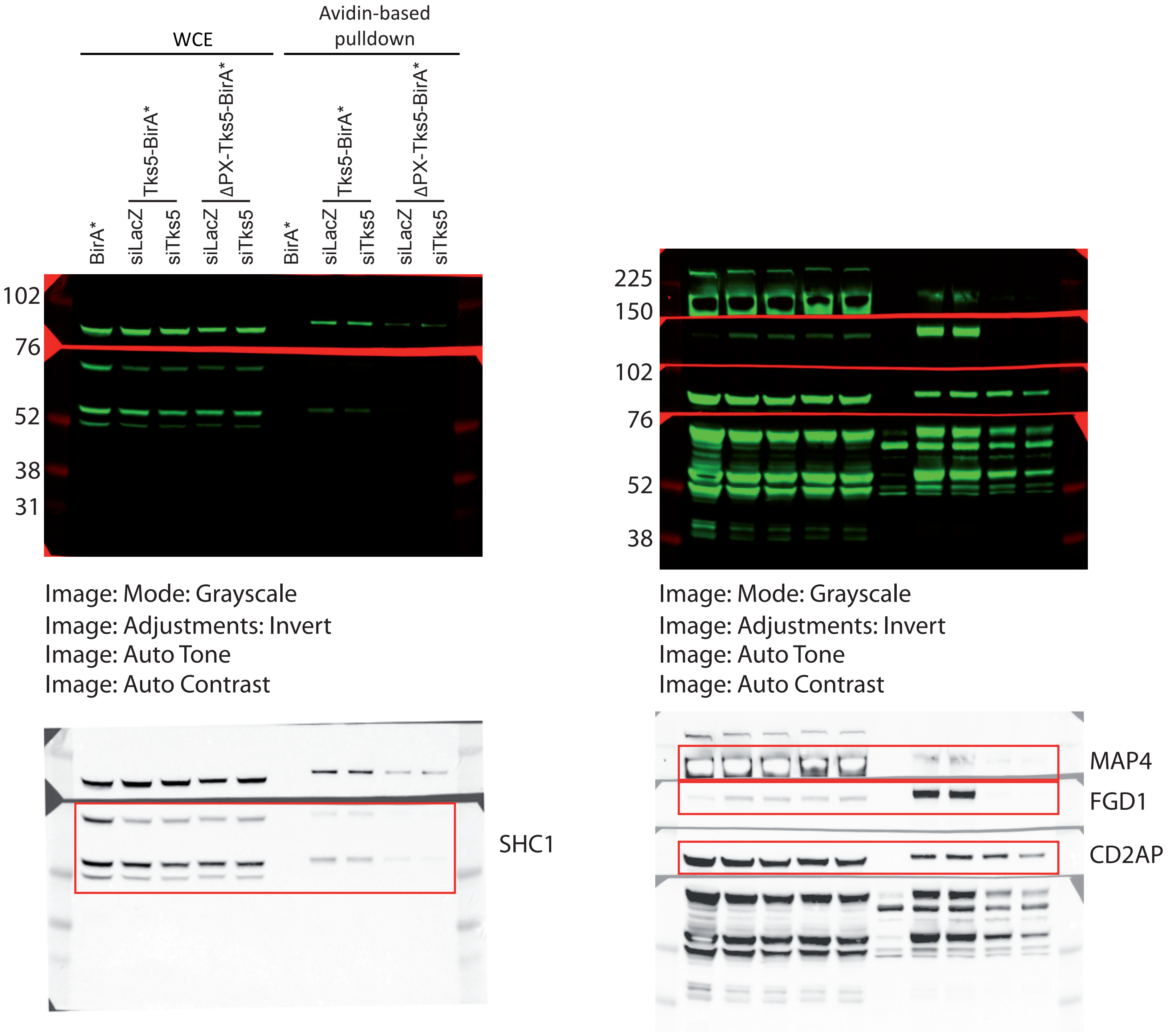

Supplement: Supplementary file 1 — Supplementary information. [file 41598_2020_63926_MOESM1_ESM.pdf]
